# Mitonuclear Interactions Produce Diverging Responses to Mild Stress in *Drosophila* Larvae

**DOI:** 10.3389/fgene.2021.734255

**Published:** 2021-09-16

**Authors:** Enrique Rodríguez, Finley Grover Thomas, M. Florencia Camus, Nick Lane

**Affiliations:** Research Department of Genetics, Evolution and Environment, University College London, London, United Kingdom

**Keywords:** mitonuclear interactions, *Drosophila melanogaster*, larvae, mitochondria, diet, high-protein, N-acetyl cysteine, oxidative stress

## Abstract

Mitochondrial function depends on direct interactions between respiratory proteins encoded by genes in two genomes, mitochondrial and nuclear, which evolve in very different ways. Serious incompatibilities between these genomes can have severe effects on development, fitness and viability. The effect of subtle mitonuclear mismatches has received less attention, especially when subject to mild physiological stress. Here, we investigate how two distinct physiological stresses, metabolic stress (high-protein diet) and redox stress [the glutathione precursor N-acetyl cysteine (NAC)], affect development time, egg-to-adult viability, and the mitochondrial physiology of *Drosophila* larvae with an isogenic nuclear background set against three mitochondrial DNA (mtDNA) haplotypes: one coevolved (WT) and two slightly mismatched (COX and BAR). Larvae fed the high-protein diet developed faster and had greater viability in all haplotypes. The opposite was true of NAC-fed flies, especially those with the COX haplotype. Unexpectedly, the slightly mismatched BAR larvae developed fastest and were the most viable on both treatments, as well as control diets. These changes in larval development were linked to a shift to complex I-driven mitochondrial respiration in all haplotypes on the high-protein diet. In contrast, NAC increased respiration in COX larvae but drove a shift toward oxidation of proline and succinate. The flux of reactive oxygen species was increased in COX larvae treated with NAC and was associated with an increase in mtDNA copy number. Our results support the notion that subtle mitonuclear mismatches can lead to diverging responses to mild physiological stress, undermining fitness in some cases, but surprisingly improving outcomes in other ostensibly mismatched fly lines.

## Introduction

Mitochondria provide most of the energy (ATP) and a significant proportion of the biosynthetic precursors and reduction potential (NADPH) needed for growth and development (Vander Heiden et al., 2009; Balsa et al., 2020). Given this centrality to development, it is hardly surprising that mitochondrial stress exercises downstream effects on signaling (Wallace and Fan, 2010; Holmstrom and Finkel, 2014), cellular differentiation (Kasahara and Scorrano, 2014), and cell death (Sweetlove et al., 2010; Vyas et al., 2016), which together impact on all aspects of fitness, health, and survival.

Mitochondrial energy transduction proceeds through the electron transfer system (ETS), generating ATP *via* oxidative phosphorylation (OXPHOS) with oxygen as the final electron acceptor (O_2_ flux). Oxygen can also react directly with FeS clusters at several sites in the ETS complexes, giving rise to reactive oxygen species (ROS flux; Quinlan et al., 2013; Pamplona et al., 2021). Once perceived as harmful by-products of mitochondrial respiration, ROS are now appreciated for their role in regulating redox tone and gene expression (Holmstrom and Finkel, 2014). Far from simply correlating with O_2_ flux, slow electron transfer through the ETS to oxygen tends to decrease ATP synthesis and increase ROS flux, as critical FeS centers become more highly reduced (Barja, 2013; Mota-Martorell et al., 2020). Slow electron transfer also impacts metabolic flux through the tricarboxylic acid (TCA) cycle, as NADH oxidation is impeded (Martínez-Reyes and Chandel, 2020). This in turn necessarily affects both growth and signaling, as TCA-cycle intermediates are key precursors for amino acid, fatty acid, nucleotide and sugar biosynthesis, as well as NADPH synthesis (Mullen et al., 2014; Bradshaw, 2019). Accumulation of TCA cycle intermediates such as succinate can induce epigenetic changes impacting on growth and development, which have been implicated in the metabolic rewiring characteristic of cancer (Deberardinis and Chandel, 2020). Clearly, anything that impairs electron transfer through the ETS could have profound effects on physiology, gene expression and fitness.

The importance of fast electron transfer for growth and development makes it all the more surprising that the respiratory complexes are mosaics of subunits encoded by two obligate cellular genomes, nuclear and mitochondrial (Blier et al., 2001; Rand et al., 2004). While the assembly and function of the respiratory complexes require precise coordination of the two genomes, there is potential for mitonuclear variation to be generated in every generation. In particular, nuclear alleles are recombined through sexual reproduction, whereas mitochondrial DNA (mtDNA) is normally inherited clonally down the maternal line, which can also produce sex-specific effects (Frank and Hurst, 1996; Gemmell et al., 2004). Equally confounding, mtDNA evolves at 10–50-fold the rate of nuclear genes (Neiman and Taylor, 2009), forcing the nuclear genome to adapt rapidly to new mitochondrial haplotypes in the population (Barreto and Burton, 2013b; Healy and Burton, 2020).

Severe incompatibilities between the mitochondrial and nuclear genomes are known to cause deleterious phenotypic effects by disturbing O_2_ and ROS fluxes. Hybrid breakdown in crosses between highly divergent populations might even contribute to speciation (Lane, 2009; Barreto and Burton, 2013a; Gershoni et al., 2014; Pichaud et al., 2019). More subtle mismatches originating from single nucleotide polymorphisms (SNPs) may not produce a phenotypic response and circulate widely in natural populations. However, even a small number of SNPs in mtDNA can perturb the expression of hundreds of nuclear genes (Innocenti et al., 2011), and these effects could be exacerbated by environmental stress (GxGxE interactions). Given that mitochondria are increasingly recognized as important drug targets for various diseases, and that adaptation to changing environments stresses mitochondrial function, there is a pressing need to better understand the molecular basis of mitonuclear interactions and their influence on life-history traits in stressful changing environments. A number of investigations have focused on the effect of mitonuclear interactions on treatments ranging from nutrition (Camus et al., 2020a) to temperature (Towarnicki and Ballard, 2017; Montooth et al., 2019) and drug responses (Villa-Cuesta et al., 2014; Santiago et al., 2021) and indeed found significant effects, but the basis of these changes at a molecular level has been little explored.

In principle, subtle mitonuclear mismatches should slow electron transfer through the ETS complexes, lowering O_2_ flux, increasing ROS flux from reduced FeS clusters, and slowing TCA cycle flux by impeding NADH oxidation (Lane, 2011; Barja, 2013; Martínez-Reyes and Chandel, 2020). Stressing mildly mismatched mitonuclear systems should amplify latent deficits and unmask new phenotypes. In this study, we probed how mitonuclear interactions modulate the response to distinct cellular stressors in a well-established *Drosophila melanogaster* model. Because larvae are constrained in their resource allocation for growth, the metabolic underpinnings of this stage in different mitonuclear genetic contexts are of particular interest. Developing larvae compete for limited food and must meet time-dependent developmental checkpoints or die (Meiklejohn et al., 2013; Rodrigues et al., 2015). Faster developing individuals have a competitive advantage, which has been linked to lower mtDNA copy numbers and higher O_2_ flux (Salminen et al., 2017). We therefore examined the responses of *Drosophila* larvae to treatments that place a strain on mitochondrial function in relation to electron transfer: the glutathione–precursor N-acetylcysteine (NAC), which interferes with ROS signaling and redox balance; and a high-protein diet, which increases TCA-cycle flux and dependence on mitochondrial respiration.

Specifically, we compared larvae from three fly lines harboring distinct mtDNAs on an isogenic nuclear background, generated through backcrosses using balancer chromosomes (Clancy, 2008). These were: wild-type (WT; w^1118-5,095^) with coevolved mtDNA and nuclear background; COX, possessing one SN*P* difference in the gene coding for the COXII subunit (Patel et al., 2016); and BAR, which has nine SNPs difference in protein-coding genes in its mtDNA, mainly in complexes I and IV (Clancy, 2008; Wolff et al., 2016). Adult BAR and COX flies have been shown to exhibit mild male subfertility at 25°C, which in the case of COX is exacerbated by higher temperatures (29°C), and accompanied by reduced complex IV activity and ROS levels (Patel et al., 2016; Camus and Dowling, 2018). Here, we show that stress does indeed amplify phenotypic differences between mitonuclear lines, and use fluorespirometry to demonstrate that these differences are largely attributable to variations in electron flux through complex I.

## Materials and Methods

### *D. melanogaster* Maintenance and Strains

All *Drosophila melanogaster* stock strains were maintained on a standard mix of molasses/cornmeal medium at a constant 25°C on a 12:12-h light–dark cycle. Three different strains of *D. melanogaster* were used in this experiment, differing only in their mitochondrial genomes. The “WT” strain was the coevolved strain, with the w^1118-5,095^ nuclear genome naturally coevolved with the mitochondrial genome. The second strain had the same isogenic w^1118-5,095^ nuclear background but had a mitochondrial haplotype termed “COXII.” The COXII haplotype was derived from *w^1118^* flies in which the mitochondrial mutation COII^G177S^ has become fixed (Patel et al., 2016). COII^G177S^ is a single non-synonymous change to subunit II of cytochrome *c* oxidase, and this SN*P* is the only difference between COXII and WT mtDNA. The third strain had a mitochondrial haplotype designated BAR and was derived from a wild population from Barcelona, Spain. For this strain, the original chromosomes were replaced by those of the *w^1118^* nuclear genome through the use of a balancer chromosome crossing scheme (Clancy, 2008). mtDNA from BAR flies differs from WT mtDNA by 9 SNPs, mostly in protein-coding genes (Wolff et al., 2016). All fly strains were kept in a strict breeding regime, whereby female flies from all strains were backcrossed to the isogenic w^1118^ nuclear background every other generation, which itself was propagated *via* full-sib crosses (also done every other generation). This regime ensured that all fly strains maintained the nuclear background as similar as possible. Freshly introgressed flies were expanded and used for this experiment, to minimize the chance of compensatory evolution happening.

Lines were propagated by 4-day-old parental flies, with approximate densities of 80–100 eggs per vial. Flies were kept at 25°C and 50% humidity, on a 12:12-h light/dark cycle, and reared on 8ml of cornmeal–molasses–agar medium per vial (see Table S1 for recipe), with *ad libitum* live yeast added to each vial to promote female fecundity. All lines had been cleared of potential bacterial endosymbionts, such as *Wolbachia*, through a tetracycline treatment at the time the lines were created. Clearance was verified using *Wolbachia*-specific PCR primers (O'Neill et al., 1992).

### Experimental Treatments

This study examined larval life-history traits and physiology across three treatments (control environment plus two experimental treatments). The first experimental treatment was exposure to the glutathione precursor (NAC - Sigma A7250). For this treatment, fly food media (control media, see Table S1) was prepared, and NAC was added at a concentration of 5mgml^−1^. More specifically, 5g of NAC was dissolved in 100ml of water and added to 900ml of liquid fly food media. Once the NAC solution and liquid media were thoroughly mixed, 4ml of NAC food was dispensed into individual fly vials. Powdered NAC and media stocks were stored at 4°C and warmed to room temperature before use.

The second experimental treatment was exposure to food medium rich in protein. Our experimental diet was formulated to have increased protein content, by increasing the amount of yeast in the diet (Table S1). The protein-to-carbohydrate ratio for this diet is approximately 1:2, whereas the control medium had a ratio of 1:4. We acknowledge that there was some variation in the nutritional components.

### Development Time and Survival Measures

Five vials of flies were collected from each haplotype within a 24-h period from eclosing and placed in vials containing *ad libitum* live yeast to boost female fecundity. When 4days of age, flies were transferred to an oviposition chamber which contained an agar–grape juice media. Adult flies were left in the oviposition chambers for 2h, and then moved onto another oviposition chamber for a further 2h (total of two clutches of egg per grou*p* of flies). We chose to setu*p* two clutches as we wanted to minimize the variance in development time stemming from timing of egg lays. Oviposition chambers were left for 24h for eggs to hatch, with the aim of collecting young first-instar larvae. We chose this methodology to avoid the possible confounding maternal effects, which could lead to inviable eggs. By picking larvae that have recently hatched we were certain that at the start point of the experiment, all offspring were alive.

Twenty first-instar larvae were picked from each clutch across all haplotype and allocated to one of the three experimental treatments (Control, NAC, Protein). Twenty vials were setu*p* for each clutch/treatment/haplotype combination. Development time was recorded as the average time it took flies from each vial to eclose. In addition to development time, we measured survival to adulthood. This assay was run over two experimental blocks differed in time by 1 generation.

### Mitochondrial Function Analysis Through High-Resolution Fluorespirometry

Simultaneous measurements of oxygen consumption and H_2_O_2_ flux in various respiratory states were performed on permeabilized third instar larvae (i.e., 6days post hatching) using an O2k-FluoRespirometer (Oroboros Instruments, Innsbruck, Austria). A substrate-uncoupler-injection-titration protocol was adapted based on the *Drosophila* thorax method by Simard et al. (2018). Because the Amplex Ultra Red (AUR) system used to detect H_2_O_2_ is incompatible with cytochrome *c,* we performed preliminary oxygen flux analyses to assess the integrity of the outer mitochondrial membrane (addition of 10μm cytochrome *c* in the N-OXPHOS state) and ensure sample quality. Two larvae from each haplotype–treatment combinations were weighed and transferred to a multi-welled plate containing 2ml of ice-cold preservation solution BIOPS (2.77mm CaK_2_EGTA, 7.23mm K_2_EGTA, 6.56mm MgCl_2_·6H_2_O, 20mm imidazole, 20mm taurine, 15mm Na_2_ phosphocreatine, 0.5mm dithiothreitol, 50mmK-MES, and 5.77mm Na_2_ATP) and 81.25μg/ml saponin for permeabilization. Larvae were then carefully opened longitudinally with tweezers and shaken at 300rpm on a plate shaker on ice for 20min, after which they were transferred to another well and rinsed for 5min in 1ml of MiR05 respirometry buffer (0.5mm EGTA, 3mm MgCl_2_.6H_2_O, 60mM lactobionic acid, 20mm taurine, 10mM KH_2_PO_4_, 20mm HEPES, 110mm D-sucrose, and 1g/l BSA, pH 7.1). This same buffer was used in the O2k-FluoRespirometer chambers, and both oxygen and fluorescence signals were calibrated as per the manufacturer’s protocols. For H_2_O_2_ analysis, 15μm DTPA, 5U/ml SOD, 1 unit HRP, and 10μm AUR were injected sequentially in the chamber prior to sample insertion. Chambers were then opened, and NADH-pathway substrates pyruvate (10mm) and malate (2mm) were added, followed by the two larvae and closing of the chambers. After 15–20min of signal stabilization in the N-LEAK state (N_L) and AD*P* (5mm) to reach the N-OXPHOS state (N_P), proline (10mm, NPro_P), succinate (10mm, NProS_P), and glycerol phosphate (10mm, NProSGp_P) were added sequentially to reach the maximum coupled respiration rates. Next, titration with the uncoupler FCC*P* in 0.5μm increments allowed estimation of the maximum uncoupled respiration (NProSGp_E). Then, the N-pathway was inhibited of with rotenone (0.5μm, ProSGp_E), the S-pathway with malonate (5mm, ProGp_E), and finally complex III with antimycin A (2.5μm), allowing the estimation of residual oxygen consumption (ROX). Chambers were opened for reoxygenation, closed before injection of ascorbate and TMPD (2.5mm and 0.5mm, respectively) to measure complex IV oxygen consumption, after which the enzyme was inhibited by the injection of 100mm of sodium azide to calculate complex IV activity (corrected for chemical background as per the manufacturer’s instructions).

Data were extracted from DatLab 7.4 software, processed using the manufacturer’s data calculation templates, and analyzed by correcting by larval weight. We calculated the respiratory control ratio (RCR) as: RCR=N_P/N_L, and substrate contributions as the fractional change in flux upon addition of the substrate (flux control efficiencies; Gnaiger, 2020). Complex I contribution was calculated as the per cent decrease in respiration following rotenone addition in the uncoupled state (ProSGp_E - NProSGp_E/NProSGp_E^*^100). The acronyms and terminology used are in accordance with the recent call for harmonization and consistency in the nomenclature of mitochondrial respiratory states and rates (Gnaiger and Group, 2020).

### mtDNA Copy Number Quantification

We collected larvae at the same timepoint respirometry would be performed (6days following hatching) and froze individual larvae across all experimental units. We extracted DNA from each individual larva using the *QIAamp DNA Micro* Kit (Qiagen, Valencia, CA) as per instruction manual. Mitochondrial copy number was measured *via* quantitative real-time PCR by amplifying a mitochondrial gene and comparing it to a single-copy nuclear gene (Correa et al., 2012); the parameter thus reflects the average number of mtDNA copies per cell (or nucleus). Mitochondrial quantification was done by amplifying a 113b*p* region of the large ribosomal subunit (CR34094 and FBgn0013686), and nuclear DNA was quantified by amplifying a 135b*p* region of the single-copy (Aoyagi and Wassarman, 2000) subunit of the RNA polymerase II gene (CG1554, FBgn0003277).

For each experimental sample, values of copy number were obtained using the following formula: 2^−ΔCt^ in which the cycle threshold ΔCt=Ct _mt_–Ct _nuc_ is a relative measure of difference between mitochondrial and nuclear gene products.

### Statistical Analyses

Larval development time and survival were analyzed using R. For development time, mitochondrial haplotype and treatment (plus their interaction) were modeled as fixed effects with development time (hours) as a response variable. For this model, we also included “clutch” as a random effect. For the survival dataset, we used a binomial general linear model with offspring and flies that failed to develop (deaths) as a response variable. Again, we used mitochondrial haplotype and treatment (plus their interaction) as fixed effects with clutch as a random effect.

Mitochondrial bioenergetic parameters obtained through fluorespirometry were analyzed by ANOVAs with type III sums of squares and Tukey’s *post hoc* tests in R (version 3.6.3) using packages *car* and *emmeans* (Fox et al., 2018; Lenth et al., 2018). Mitochondrial haplotype and treatment (and their interaction) were the fixed effects, and the various mitochondrial parameters (specific O_2_ and H_2_O_2_ fluxes at each respiratory state and FCRs) were the response variables.

Copy number variation was modeled using a linear model, with copy number as a response variable and mitochondrial haplotype and treatment (plus their interaction) as fixed effects. Models were implemented using the *lm* and *Anova* functions in R. For further analysis of the data, we used Tukey’s *post hoc* tests implemented in the *emmeans* package in R (Lenth et al., 2018).

## Results

### Development Time and Survival

**Figure 1:**
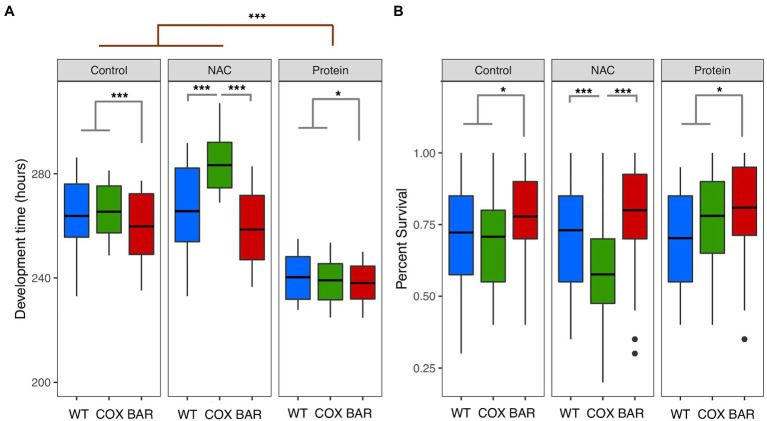
Development time **(A)** and percent survival **(B)** for larvae of the three mitochondrial haplotypes (WT, COX, and BAR) across the three environmental conditions. Asterisks denote significant differences (^*^p < 0.05, ^***^p < 0.001).

We first found a significant mitochondrial effect (*F* = 118.162, *p* < 0.001, Figure 1A) across all treatments, where flies harboring the BAR haplotype had a faster development time than both WT and COX. We also found a significant treatment effect (*F* = 710.99, *p* < 0.001, Figure 1A), with the high protein treatment decreasing development time across all haplotypes. Moreover, we detected a significant mitochondria-by-treatment interaction, indicating a more complex dynamic in our results. This result was largely driven by COX flies being significantly impacted by the NAC treatment, having a very slow development (Tukey’s *post hoc*: WT_N_-COX_N_, *p* < 0.001; BAR_N_-COX_N_, *p* < 0.001, Figure 1A).

While we did not find an overall effect of treatment on survival (*χ*^2^ = 0.881, *p* = 0.643, Figure 1B), we found a significant interaction between mitochondrial haplotype and treatment (*χ*^2^ = 32.166, *p* < 0.001, Figure 1B). This interaction was driven by the decreased survival of the COX haplotype on NAC food (Tukey’s *post hoc*: WT_N_-COX_N_, *p* < 0.001; BAR_N_-COX_N_, *p* < 0.001, Figure 1B). We also found that across all treatments, the BAR haplotype had increased proportion survival compared to the other two haplotypes (*χ*^2^ = 17.802, *p* < 0.001, Figure 1B).

### Mitochondrial Function

**Figure 2:**
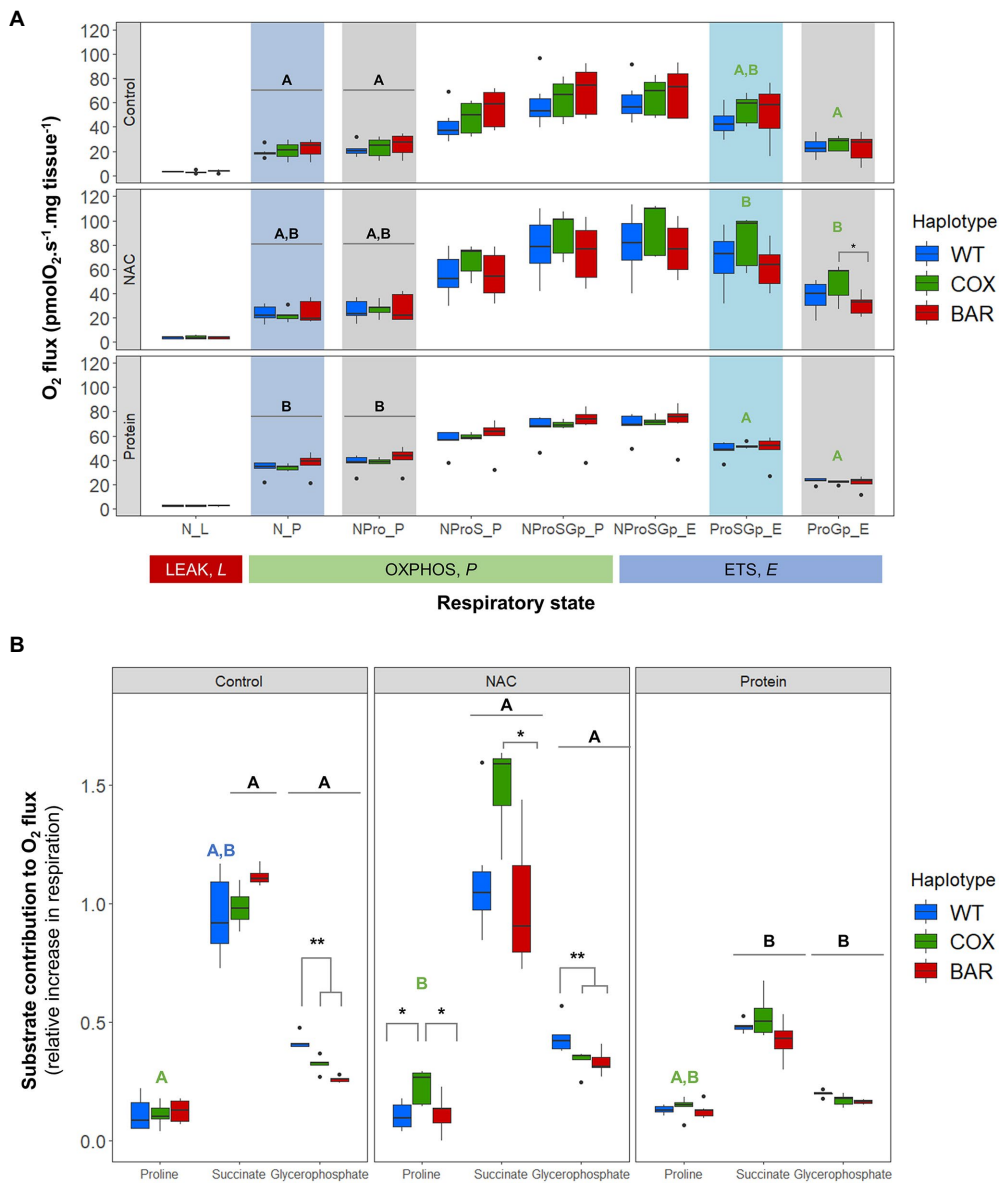
Mitochondrial function in third-instar *D. melanogaster* larvae. **(A)** Oxygen flux normalized by larvae wet weight as a function of respiratory state in the three mitochondrial haplotypes (WT, COX, and BAR) grown under control, NAC, and protein treatments. **(B)** Contributions of substrates proline, succinate and glycerophosphate to respiration, measured as the increment in O_2_ flux from the previous respiratory state. Acronyms refer to leak (L), OXPHOS (P), and uncoupled ETS (E) states and to the substrates used in the various steps (N, NADH-pathway substrates pyruvate and malate; Pro, proline; S, succinate; Gp, glycerophosphate). Boxplots depict median values for each haplotype and treatment (*n* = 5 to 7), 25th and 75th percentiles, inter-quartile range, and outliers. Colored states and letters reflect where significant differences (*p* < 0.05) were detected between treatments for a given respiratory state and mitochondrial haplotype. Asterisks show significant differences (^*^p < 0.05, ^**^p < 0.01) between haplotypes for a given treatment.

Figure 2A shows oxygen consumption in control and treatment larvae as a function of respiratory state. We found significant effects of treatment on the O_2_ flux (normalized by larvae wet weight in mg tissue) in various respiratory states, contingent on haplotype (see Supplementary Material). The OXPHOS state with N-pathway substrates (pyruvate and malate), N_P, was influenced by treatment (*F* = 20.037, *p* < 0.001, Figure 2), with protein-treated O_2_ flux being higher than control in all haplotypes (Tukey’s *post hoc*: WT_C_-WT_P_, *p* = 0.037; COX_C_-COX_P_, *p* = 0.0481; BAR_C_-BAR_P_, *p* = 0.021). Similarly, the addition of proline in NPro_*P* showed a significant effect of treatment (*F* = 18.160, *p* < 0.001, Figure 2) and a higher O_2_ flux in protein-treated larvae compared to controls in all haplotypes (Tukey’s *post hoc*: WT_C_-WT_P_, *p* = 0.049; COX_C_-COX_P_, *p* = 0.048; BAR_C_-BAR_P_, *p* = 0.0376). In the ETS (uncoupled) state and after rotenone inhibition (ProSGp_E), there was a significant effect of treatment (*F* = 9.836, *p* < 0.001, Figure 2), with COX flies on NAC having a higher O_2_ flux compared to protein (Tukey’s *post hoc*: COX_N_-COX_P_, *p* = 0.0485). Similarly, there was a significant effect of treatment on malonate-induced ProGp_E flux (*F* = 17.570, *p* < 0.001, Figure 2A), with the NAC treatment having a higher O_2_ flux than control and protein (Tukey’s *post hoc*: COX_C_-COX_N_, *p* = 0.007; COX_N_-COX_P_, *p* < 0.001). Moreover, the O_2_ flux for NAC-treated COX flies was significantly higher than in NAC-treated BAR larvae in this respiratory state (Tukey’s *post hoc*: COX_N_-BAR_N_, *p* = 0.025).

**Figure 3:**
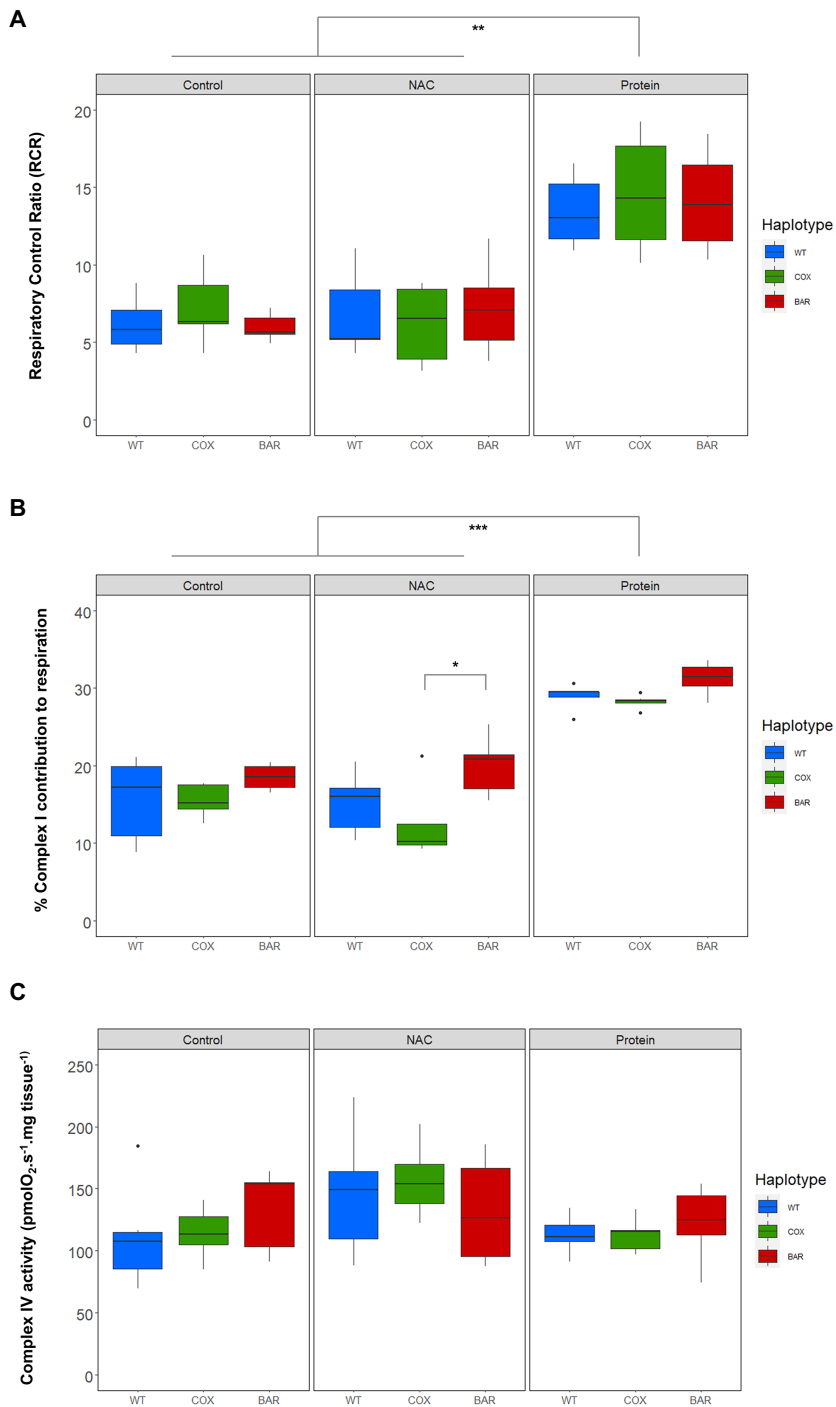
Parameters of mitochondrial function in the three mitochondrial haplotypes (WT, COX, and BAR) of third-instar *D. melanogaster* larvae grown under control, NAC and protein treatments. **(A)** Respiratory control ratio (RCR) corresponding to states N_P/N_L (with NADH-pathway substrates pyruvate and malate). **(B)** Complex I contribution measured as the % decrease in respiration following rotenone addition in the E-state. **(C)** Complex IV activity measured with the ascorbate–TMPD assay and corrected for autooxidation. Boxplots depict median values for each haplotype and treatment (*n* = 5 to 7), 25th and 75th percentiles, inter-quartile range, and outliers. Barplots show median values (±S.E.M.) for each haplotype and treatment (*n* = 5 to 7). Colored states and letters reflect where significant differences (*p* < 0.05) were detected between treatments for a given substrate and mitochondrial haplotype. Asterisks show significant differences (^*^p < 0.05, ^**^p < 0.01, ^***^p < 0.001) between haplotypes for a given treatment.

We calculated respiratory control ratios (RCR) for each haplotype–treatment combination, defined as OXPHOS (N_P) over leak respiration (N_L), and found a significant effect of treatment (*F* = 44.055, *p* < 0.001, Figure 3A), with the protein treatment significantly increasing the RCR in all haplotypes compared to control and NAC (Tukey’s *post hoc*: WT_C_-WT_P_, *p* = 0.0014; COX_C_-COX_P_, *p* = 0.0014; BAR_C_-BAR_P_, *p* = 0.0003; WT_N_-WT_P_, *p* = 0.0043; COX_N_-COX_P_, *p* = 0.0002; BAR_N_-BAR_P_, *p* = 0.0009). We then analyzed the contribution of each substrate to O_2_ flux. We found a significant effect of the interaction between haplotype and treatment on the contribution of proline to O_2_ flux (*F* = 2.657, *p* = 0.046, Figure 3B), which was higher in NAC-treated than control COX larvae (Tukey’s *post hoc*: COX_C_-COX_N_, *p* = 0.042). There were also differences in haplotype response to NAC, with proline contribution in COX being higher than in WT and BAR (Tukey’s *post hoc*: WT_N_-COX_N_, *p* = 0.019; COX_N_-BAR_N_, *p* = 0.028). The contribution of succinate to O_2_ flux also showed a significant effect of the interaction between haplotype and treatment (*F* = 2.726, *p* = 0.042, Figure 3B) and of treatment alone (*F* = 42.604, *p* < 0.001). In particular, all haplotypes had a lower contribution of succinate in the protein treatment compared to NAC and control, except in WT where it was only significantly different from NAC (Tukey’s *post hoc*: WT_C_-WT_P_, *p* = 0.081; WT_N_-WT_P_, *p* = 0.005; COX_C_-COX_P_, *p* = 0.003; COX_N_-COX_P_, *p* < 0.001; BAR_C_-BARP, *p* < 0.001; BAR_N_-BAR_P_, *p* = 0.005). Within the NAC treatment, succinate contribution was higher in COX than BAR larvae (Tukey’s *post hoc*: COX_N_-BAR_N_, *p* = 0.043). As for glycerophosphate (Gp) contribution to O_2_ flux, we found a significant effect of haplotype (*F* = 35.820, *p* < 0.001), treatment (*F* = 112.694, *p* < 0.001), and their interaction (*F* = 3.770, *p* = 0.010) with various differences among haplotypes (Tukey’s *post hoc*: WT_C_-WT_P_, *p* < 0.001; WT_N_-WT_P_, *p* < 0.001, COX_C_-COX_P_, *p* < 0.001; COX_N_-COX_P_, *p* < 0.001; BAR_C_-BAR_P_, *p* = 0.007; BAR_N_-BAR_P_, *p* < 0.001; WT_C_-COX_C_, *p* = 0.009; WT_C_-BAR_C_, *p* < 0.001; WT_N_-COX_N_, *p* = 0.003, WT_N_-BAR_N_, *p* < 0.001). The contribution of G*p* to respiration was lower in the protein-treated larvae than in the control and NAC, and within these two, WT relied more heavily on G*p* than COX and BAR.

The contribution of complex I to respiration, measured as the per cent decrease in respiration following rotenone addition in the E-state, showed significant mitochondrial (*F* = 8.560, *p* < 0.001, Figure 3C) and treatment effects (*F* = 89.462, *p* < 0.001). Protein treatment significantly increased complex I contribution compared to control and NAC (Tukey’s *post hoc*: WT_C_-WT_P_, *p* < 0.001; WT_N_-WT_P_, *p* < 0.001, COX_C_-COX_P_, *p* < 0.001; COX_N_-COX_P_, *p* < 0.001; BAR_C_-BAR_P_, *p* = 0.007; BAR_N_-BAR_P_, *p* < 0.001), while BAR_N_ maintained a high complex I contribution compared to COX (Tukey’s *post hoc*: COX_N_-BAR_N_, *p* = 0.015). We found a significant treatment effect in the measure of complex IV activity (*F* = 3.561, *p* = 0.037, Figure 3C), but no subsequent significant pairwise comparisons among haplotypes and treatments.

**Figure 4:**
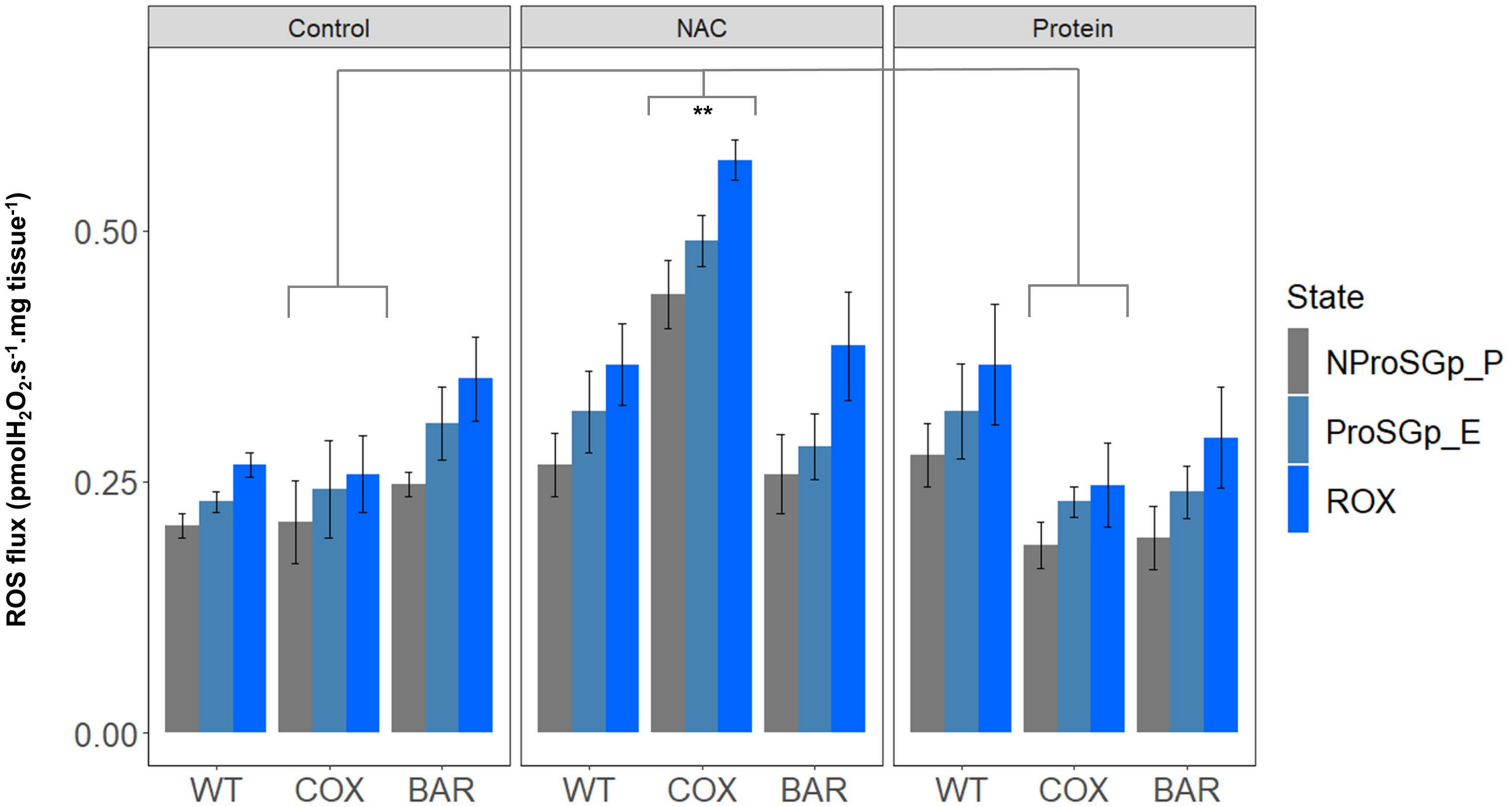
Reactive oxygen species flux (measured as H_2_O_2_ flux) across the three haplotype (WT, COX, and BAR) and treatments studied (control, NAC, and protein), normalized by larvae wet weight. Barplots show fluxes in maximum state 3 respiration with pyruvate, malate, proline, succinate and glycerophosphate (NProSGp_P, grey); rotenone inhibition (ProSGp_E, light blue); and with maximal inhibition with malonate and antimycin A (ROX, blue). Median values (±S.E.M) are depicted for each haplotype and treatment (*n* = 5 to 7). Asterisks show significant differences (^**^p < 0.01) between treatments.

When measuring H_2_O_2_ flux per mg tissue in the NProSGp_*P* (OXPHOS respiration with all the substrates), we found significant effects of the interaction between haplotype and treatment (*F* = 3.363, *p* = 0.026, Figure 4), as well as of treatment only (*F* = 7.908, *p* = 0.002), where flies of the COX haplotype had a higher flux on NAC than on control and protein treatments (Tukey’s *post hoc*: COX_C_-COX_N_, *p* = 0.008; COX_N_-COX_P_, *p* = 0.005). In the rotenone-induced H_2_O_2_ flux (ProSGp_E), we found a significant effect of both the interaction between haplotype and treatment (*F* = 5.597, *p* = 0.003, Figure 4) and treatment alone (*F* = 7.330, *p* = 0.003), again with a higher effect in flies with the COX haplotype on NAC (Tukey’s *post hoc*: COX_C_-COX_N_, *p* = 0.003; COX_N_-COX_P_, *p* = 0.003). In the state eliciting the highest H_2_O_2_ flux, i.e., inhibition with rotenone, malonate, and antimycin A (ROX), we also found an effect of both fixed terms (*F* = 4.271, *p* = 0.010, Figure 4) and treatment (*F* = 9.416, *p* = 0.001), although this was contingent on the haplotype (Tukey’s *post hoc*: COX_C_-COX_N_, *p* = 0.003; COX_N_-COX_P_, *p* = 0.003).

### Mitochondrial Copy Number Variation

**Figure 5:**
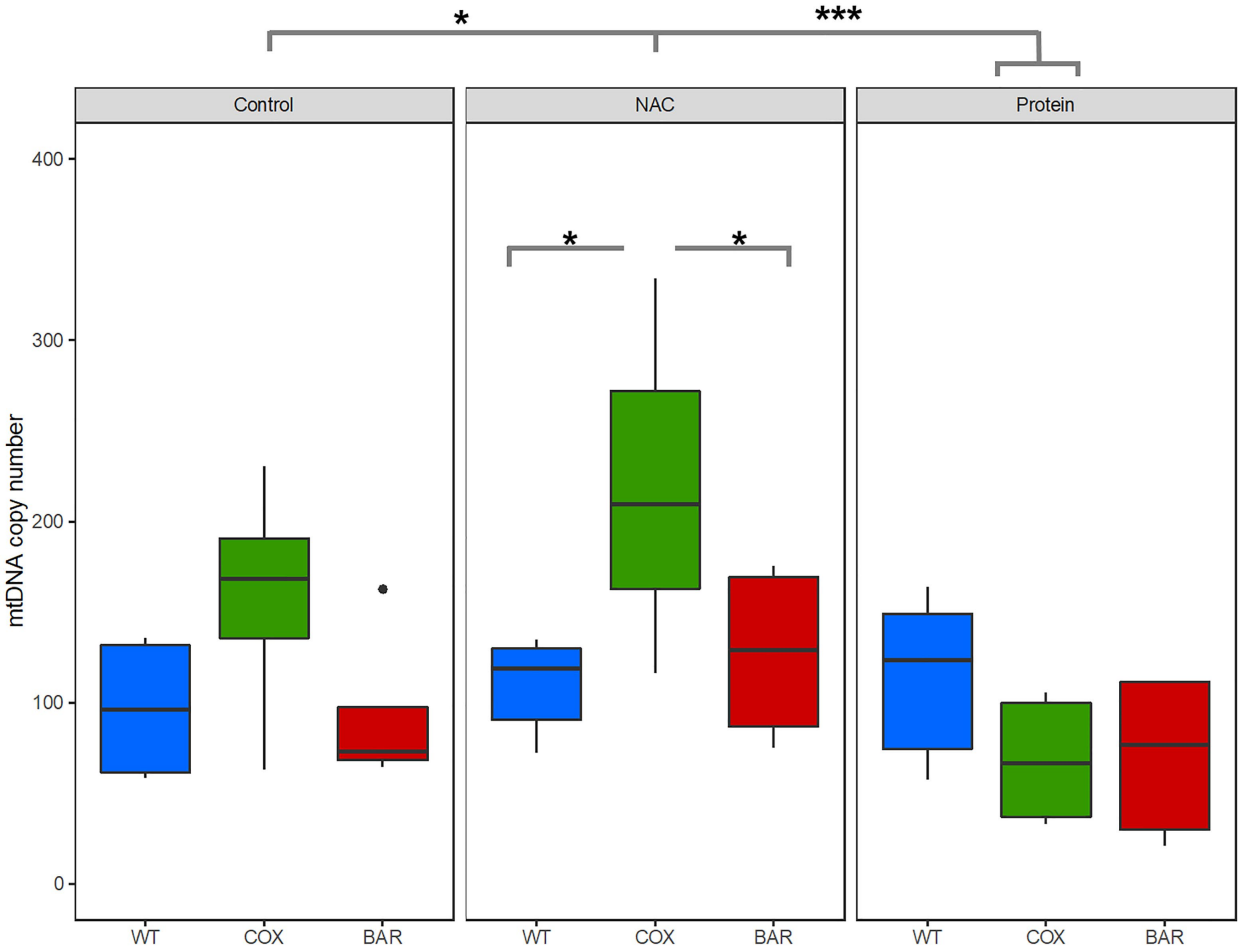
Mitochondrial DNA copy number variation across all three mitochondrial haplotypes and environmental treatments. Asterisks denote significant differences (^*^p < 0.05, ^***^p < 0.001).

For copy number variation, we found a significant mito-by-treatment interaction (*F* = 2.8662, *p* = 0.040778, Figure 5). Further investigation using *post hoc* analyses revealed that the interaction was driven by a significant increase in copy number for flies carrying the COX haplotype when exposed to NAC (Tukey’s *post hoc*: WT_N_-COX_N_, *p* = 0.0105; BAR_N_-COX_N_, *p* = 0.0477). We also found a significant decrease in copy number in protein-treated COX flies, compared to control and NAC (Tukey’s *post hoc*: COX_C_-COX_P_, *p* = 0.0480; COX_N_-COX_P_, *p* = 0.0006).

## Discussion

Serious incompatibilities between mitochondrial and nuclear genes, perhaps generated through introgression between divergent populations in changing environments, can cause hybrid breakdown and even speciation, with severe effects on mitochondrial physiology undermining fitness and survival (Wolff et al., 2014). However, the effects of more subtle mitonuclear mismatches, generated through sex within populations, on responses to mild stress are harder to predict. For example, subtle mitonuclear mismatches may generate a hormetic response that protects against the stress, while selection for mitochondrial function in specific environments (for example in relation to diet or temperature) could potentially offset the effects of subtle mismatches. Less work has been done on these ‘covert’ GxGxE interactions, yet their very unpredictability makes them potentially important in relation to both adaptation to changing environments and to health and personalized medicine. Because growth arguably places the greatest stress on all-round mitochondrial function (requiring balanced ATP synthesis, reducing equivalents in the form of NADPH and biosynthetic precursors), we considered the effects of mild stress on the development of *Drosophila* larvae. Specifically, we considered the effect of metabolic stress (a high-protein diet known to shorten the lifespan of adult *Drosophila*, Camus et al., 2020b) and redox stress (the glutathione precursor NAC) on developmental time, survival, and underlying mitochondrial function in one coevolved and two slightly mismatched *Drosophila* larvae lines. We show that mitonuclear interactions do indeed substantively influence responses to stress, with one mismatched line (COX) faring especially badly in response to redox stress, and another, BAR, consistently outperforming the coevolved WT line, despite the nuclear background being isogenic in all three lines.

When developing on control diet (standard molasses preparation), BAR flies developed significantly faster, had higher survival and tended to have a greater O_2_ flux than the other two lines (Figures 1, 2A). The underlying mitochondrial phenotype showed no difference in their respiratory control ratio under control conditions (Figure 3A), but there was less reliance on glycerol phosphate as a substrate for respiration (through GpDH feeding electrons into the ubiquinone pool, Figure 2B) than the coevolved line. This hints at a higher contribution of complex I (nonsignificant, Figure 3B), and a lower (albeit nonsignificant) mtDNA copy number (Figure 5), a suggestion that was unmasked in response to stress. When fed the redox stressor NAC, BAR larvae maintained their high complex I contribution, whereas the complex I contribution of COX larvae fell to little more than half that of BAR larvae (Figure 3B). The robust complex I-driven respiration in BAR larvae could be linked with SNPs in complex I subunits ND4L and ND5, which have previously been associated with adaptive responses to climate in birds and humans (Balloux et al., 2009; Van Der Heijden et al., 2019). We note that the mean summer temperatures in Barcelona are 5–8°C greater than Oregon, imposing greater metabolic demands on BAR mitochondria (as the Q_10_ suggests a doubling in metabolic rate for every 10°C rise in temperature). While the Oregon strain was collected in 1925, hence has had nearly a century to adapt to lab conditions, differences in heat tolerance at the species level can persist for decades in the lab, and correspond to differences in complex I and substrate use (Jørgensen et al., 2019, 2021). It is possible that BAR flies could have a respiratory architecture adapted to higher temperatures. Our results suggest that higher metabolic rates could be sustained by selection for the fastest and most efficient coupling of electron transfer to proton pumping *via* complex I-linked respiration, notably the rapid removal and oxidation of reduced ubiquinone from complex I. If so, then BAR flies may be better adapted to metabolic and redox stress than COX or WT flies due to climatic differences between their sites of origin, which outweigh the effects of mismatching mtDNA against the isogenic nuclear background; but whatever the reason, it is unequivocal that BAR flies have more robust complex I-linked respiration.

In contrast, COX larvae were especially vulnerable to redox stress. We used a NAC concentration in the mid-range of an earlier study (Brack et al., 1997) showing some lifespan benefits (at 1 and 10mg.ml^−1^), and found a strong haplotype-specific response: WT and BAR were little affected by NAC in any parameters studied, whereas COX larvae had significantly slower development, lower survival, metabolic rewiring away from complex I-linked substrates, elevated ROS flux, and raised copy number of mtDNA. NAC appeared to cause oxidative stress associated with increased reliance on alternative substrates, notably succinate (which primarily feeds electrons into complex II) and proline (where ProDH transfers electrons to the ubiquinone pool and complex III (McDonald et al., 2018). ProDH is a recognized source of ROS production, mediating mitochondrial apoptosis and tumor growth (Kononczuk et al., 2015; Soares et al., 2015). ROS signaling in COX larvae was also perturbed, as illustrated by the high H_2_O_2_ flux in the maximal OXPHOS state and during inhibition of complexes I, II, and III (Figure 4). mtDNA copy number was also significantly raised in COX larvae on NAC compared with the other two haplotypes. This suggests a hormetic response, in which mitochondrial biogenesis partially offset the respiratory deficiency (Taylor and Turnbull, 2005; Lane, 2011) but was unable to fully protect against redox stress.

The seemingly paradoxical increase in ROS production produced by replenishing the matrix antioxidant glutathione using NAC is commonly referred to as reductive stress (Samuni et al., 2013; Korge et al., 2015). Slow electron transfer through complex I can drive reductive stress, as NADH oxidation is hindered, impacting on TCA-cycle flux and the regeneration of mitochondrial NADPH. Shifts in the NADH/NAD^+^ and NADPH/NADP^+^ ratios, as well as flavin reduction in the ETS, become the key factors determining the rate of ROS production (Korge et al., 2015). In COX larvae, the single SN*P* difference in the COXII subunit of complex IV has been shown to slow electron transfer at higher temperatures, when metabolic demands are greater (Patel et al., 2016). CIV passes on its electrons directly to oxygen, so it is not surprising that it exerts significant control over the overall rates of electron flow (Rodriguez et al., 2021). While the maximal rates of both coupled and uncoupled respiration were similar in COX larvae and the other fly lines (Figure 2B)—as well as complex IV activity itself (Figure 3C)—the shift in substrate usage and respiratory architecture plainly drove reductive stress when subjected to mild redox stress from NAC. This finding has critical ramifications for adaptation to stressful environments or pharmacological treatments, as the nuclear background of COX, WT, and BAR larvae are all isogenic. The only differences that we could measure—which had pervasive effects on development—were when COX larvae were mildly stressed with an antioxidant.

Feeding larvae with a high-protein diet consistently promoted faster development in the larvae of all three haplotypes, with BAR larvae once again outperforming the other lines in terms of increased survival and faster development (Figure 1). Compared with control and NAC-treated larvae, all protein-fed larvae had higher coupled O_2_ flux on N-pathway substrates (Figure 2A), lower reliance on succinate and G*p* pathways (Figure 2B), greater RCR (Figure 3A), and higher flux through complex I (Figure 3B). Curiously, the range values for virtually all respiratory parameters (Figure 2) were consistently smaller in protein-fed larvae, which might reflect tighter constraints on permissible respiratory architecture. *Drosophila* larvae have previously been reported to be more complex I-dependent than adults, which tend to rely more on complex III-linked substrates (Ballard and Youngson, 2015), potentially explaining the accelerated growth of BAR larvae. While ROS flux was not significantly impacted by protein treatment in WT and BAR flies (Figure 4), mtDNA copy number decreased in protein-treated COX compared with NAC and control larvae (Figure 5). This suggests a compensatory decrease in mitogenesis in COX larvae on this treatment, linked with lower ROS production. It is striking that ‘forcing’ COX larvae to increase flux through complex I was beneficial in terms of developmental time, survival, all respiratory parameters, ROS flux, and mtDNA copy number. From these results in larvae, it is interesting to contemplate why a high-protein diet should substantially decrease adult lifespan (Camus et al., 2020b), given that larval growth would seem to maximize demands on resource allocation.

Proteins are broken down into their amino acid constituents, notably glutamine. This is deaminated into glutamate and enters the mitochondria *via* the glutamate–aspartate carrier (Gnaiger, 2020). Glutamate is an anaplerotic substrate, which feeds into the TCA cycle at α-ketoglutarate to regenerate NADH, and hence support complex I respiration (Gnaiger and Group, 2020). Deficiencies in electron transfer through complex I increase the likelihood of reverse TCA-cycle flux, as increases in the NADH/NAD^+^ and α-ketoglutarate to citrate ratios stimulate reductive glutamine metabolism and ultimately lipid and lactate accumulation (Ballard and Youngson, 2015). Glutamine also regulates the mammalian target of rapamycin (mTOR) pathway promoting cellular growth (Altman et al., 2016). In diseases caused by complex I mutations, high-protein diets can exacerbate ROS production (Ballard, 2016) and could upregulate the mTOR axis, potentially driving quasi-programs linked with hyperfunction and diminished stress resistance (Blagosklonny, 2013; Wang et al., 2018). While we did not observe complex I defects in larvae exposed to high-protein treatment, it may be that damage to complex I later in adult life tends to drive reverse TCA flux, promoting an age-related growth phenotype that shortens lifespan in adult flies. Be that as it may, our results suggest that robust complex I function is indeed central to larval development and survival.

In conclusion, we report that the response of *Drosophila* larvae to mild metabolic or redox stress is strongly contingent on mitonuclear interactions. Ostensibly benign differences that do not manifest phenotypically in a standard rearing environment produce unpredictable outcomes depending on the type of stress and the mitonuclear background in question. Both NAC and high-protein treatment primarily affected flux at complex I, but the metabolic and phenotypic consequences were very different depending on the haplotype. Our study highlights the need to understand how subtle differences in mitonuclear interactions, amplified by stress, manifest through the rewiring of metabolic flux, signaling, gene expression and ultimately phenotype. These pervasive and fundamental effects are likely to hold important implications for health (personalized medicine) and biodiversity (adaptation and speciation) in a world where climate change will surely amplify mitonuclear stress.

## Data Availability Statement

All data are available on the Figshare Digital Repository; doi: 10.5522/04/16539723

## Author Contributions

ER, MC, and NL conceived the experiment. FG, ER, and MC collected and analyzed the data. All authors contributed to writing the manuscript.

## Funding

This research was funded by the BBSRC (BB/S003681/1) and Leverhulme Trust (RPG-2019-109) grants to NL and MC.

## Conflict of Interest

The authors declare that the research was conducted in the absence of any commercial or financial relationships that could be construed as a potential conflict of interest.

## Publisher’s Note

All claims expressed in this article are solely those of the authors and do not necessarily represent those of their affiliated organizations, or those of the publisher, the editors and the reviewers. Any product that may be evaluated in this article, or claim that may be made by its manufacturer, is not guaranteed or endorsed by the publisher.

## References

[ref1] Altman B. J. Stine Z. E. Dang C. V. (2016). From Krebs to clinic: glutamine metabolism to cancer therapy. Nat. Rev. Cancer 16:749. doi: 10.1038/nrc.2016.114, PMID: 28704361

[ref2] Aoyagi N. Wassarman D. A. (2000). Genes encoding Drosophila melanogaster RNA polymerase II general transcription factors: diversity in TFIIA and TFIID components contributes to gene-specific transcriptional regulation. J. Cell Biol. 150, F45–F50. doi: 10.1083/jcb.150.2.F45, PMID: 10908585 PMC2180226

[ref3] Ballard J. (2016). (2016). Can we alter dietary macronutrient compositions and alleviate mitochondrial disease. J. Rare Dis. Res. Treat. 1, 31–37. doi: 10.29245/2572-9411/2016/3.1043

[ref4] Ballard J. W. Youngson N. A. (2015). Review: can diet influence the selective advantage of mitochondrial DNA haplotypes? Biosci. Rep. 35:e00277. doi: 10.1042/BSR20150232, PMID: 26543031 PMC4708006

[ref5] Balloux F. Handley L.-J. L. Jombart T. Liu H. Manica A. (2009). Climate shaped the worldwide distribution of human mitochondrial DNA sequence variation. Proc. R. Soc. B Biol. Sci. 276, 3447–3455. doi: 10.1098/rspb.2009.0752, PMID: 19586946 PMC2817182

[ref6] Balsa E. Perry E. A. Bennett C. F. Jedrychowski M. Gygi S. P. Doench J. G.. (2020). Defective NADPH production in mitochondrial disease complex I causes inflammation and cell death. Nat. Commun. 11:2714. doi: 10.1038/s41467-020-16423-1, PMID: 32483148 PMC7264245

[ref7] Barja G. (2013). Updating the mitochondrial free radical theory of aging: an integrated view, key aspects, and confounding concepts. Antioxid. Redox Signal. 19, 1420–1445. doi: 10.1089/ars.2012.5148, PMID: 23642158 PMC3791058

[ref8] Barreto F. S. Burton R. S. (2013a). Elevated oxidative damage is correlated with reduced fitness in interpopulation hybrids of a marine copepod. Proc. R. Soc. B Biol. Sci. 280:20131521. doi: 10.1098/rspb.2013.1521, PMID: 23902912 PMC3735265

[ref9] Barreto F. S. Burton R. S. (2013b). Evidence for compensatory evolution of ribosomal proteins in response to rapid divergence of mitochondrial rRNA. Mol. Biol. Evol. 30, 310–314. doi: 10.1093/molbev/mss228, PMID: 22993236

[ref10] Blagosklonny M. V. (2013). Aging is not programmed: genetic pseudo-program is a shadow of developmental growth. Cell Cycle 12, 3736–3742. doi: 10.4161/cc.27188, PMID: 24240128 PMC3905065

[ref11] Blier P. Dufresne F. Burton R. S. (2001). Natural selection and the evolution of mtDNA-encoded peptides: evidence for intergenomic co-adaptation. Trends Genet. 17, 400–406. doi: 10.1016/S0168-9525(01)02338-1, PMID: 11418221

[ref12] Brack C. Bechter-Thuring E. Labuhn M. (1997). N-acetylcysteine slows down ageing and increases the life span of Drosophila melanogaster. Cell. Mol. Life Sci. 53, 960–966. doi: 10.1007/pl00013199, PMID: 9447249 PMC11147307

[ref13] Bradshaw P. (2019). Cytoplasmic and mitochondrial NADPH-coupled redox Systems in the Regulation of aging. Nutrients 11:504. doi: 10.3390/nu11030504, PMID: 30818813 PMC6471790

[ref14] Camus M. F. Dowling D. K. (2018). Mitochondrial genetic effects on reproductive success: signatures of positive intrasexual, but negative intersexual pleiotropy. Proc. R. Soc. B Biol. Sci. 285:20180187. doi: 10.1098/rspb.2018.0187, PMID: 29794041 PMC5998096

[ref15] Camus M. F. Moore J. Reuter M. (2020a). Nutritional geometry of mitochondrial genetic effects on male fertility. Biol. Lett. 16:20190891. doi: 10.1098/rsbl.2019.0891, PMID: 32097597 PMC7058949

[ref16] Camus M. F. O'leary M. Reuter M. Lane N. (2020b). Impact of mitonuclear interactions on life-history responses to diet. Philos. Trans. R. Soc. B 375:20190416. doi: 10.1098/rstb.2019.0416, PMID: 31787037 PMC6939373

[ref17] Clancy D. J. (2008). Variation in mitochondrial genotype has substantial lifespan effects which may be modulated by nuclear background. Aging Cell 7, 795–804. doi: 10.1111/j.1474-9726.2008.00428.x, PMID: 18727704

[ref18] Correa C. C. Aw W. C. Melvin R. G. Pichaud N. Ballard J. W. (2012). Mitochondrial DNA variants influence mitochondrial bioenergetics in Drosophila melanogaster. Mitochondrion 12, 459–464. doi: 10.1016/j.mito.2012.06.005, PMID: 22735574

[ref19] Deberardinis R. J. Chandel N. S. (2020). We need to talk about the Warburg effect. Nat. Metab. 2, 127–129. doi: 10.1038/s42255-020-0172-2, PMID: 32694689

[ref20] Fox J. Weisberg S. Price B. Adler D. Bates D. Baud-Bovy G. (2018). Car: Companion to Applied Regression. R package version 3.0–2. Software. Available at: https://cran.r-project.org/web/packages/car (Accessed June 1, 2018).

[ref21] Frank S. A. Hurst L. D. (1996). Mitochondria and male disease. Nature 383:224. doi: 10.1038/383224a0, PMID: 8805695

[ref22] Gemmell N. J. Metcalf V. J. Allendorf F. W. (2004). Mother's curse: the effect of mtDNA on individual fitness and population viability. Trends Ecol. Evol. 19, 238–244. doi: 10.1016/j.tree.2004.02.002 16701262

[ref23] Gershoni M. Levin L. Ovadia O. Toiw Y. Shani N. Dadon S.. (2014). Disrupting mitochondrial–nuclear coevolution affects OXPHOS complex I integrity and impacts human health. Genome Biol. Evol. 6, 2665–2680. doi: 10.1093/gbe/evu208, PMID: 25245408 PMC4224335

[ref24] Gnaiger E. (2020). Mitochondrial pathways and respiratory control. An introduction to OXPHOS analysis. Bioenerg. Commun. 2:112. doi: 10.26124/bec:2020-0002

[ref25] Gnaiger E. Group, M.T (2020). “Mitochondrial physiology,” in Bioenergetics Communications. MiPsociety.

[ref26] Healy T. M. Burton R. S. (2020). Strong selective effects of mitochondrial DNA on the nuclear genome. Proc. Natl. Acad. Sci. U. S. A. 117, 6616–6621. doi: 10.1073/pnas.1910141117, PMID: 32156736 PMC7104403

[ref27] Holmstrom K. M. Finkel T. (2014). Cellular mechanisms and physiological consequences of redox-dependent signalling. Nat. Rev. Mol. Cell Biol. 15, 411–421. doi: 10.1038/nrm3801, PMID: 24854789

[ref28] Innocenti P. Morrow E. H. Dowling D. K. (2011). Experimental evidence supports a sex-specific selective sieve in mitochondrial genome evolution. Science 332, 845–848. doi: 10.1126/science.1201157, PMID: 21566193

[ref29] Jørgensen L. B. Malte H. Overgaard J. (2019). How to assess drosophila heat tolerance: unifying static and dynamic tolerance assays to predict heat distribution limits. Funct. Ecol. 33, 629–642. doi: 10.1111/1365-2435.13279

[ref30] Jørgensen L. B. Overgaard J. Hunter-Manseau F. Pichaud N. (2021). Dramatic changes in mitochondrial substrate use at critically high temperatures: a comparative study using drosophila. J. Exp. Biol. 224:jeb240960. doi: 10.1242/jeb.240960, PMID: 33563650

[ref31] Kasahara A. Scorrano L. (2014). Mitochondria: from cell death executioners to regulators of cell differentiation. Trends Cell Biol. 24, 761–770. doi: 10.1016/j.tcb.2014.08.005, PMID: 25189346

[ref32] Kononczuk J. Czyzewska U. Moczydlowska J. Surażyński A. Palka J. Miltyk W. (2015). Proline oxidase (POX) as A target for cancer therapy. Curr. Drug Targets 16, 1464–1469. doi: 10.2174/138945011613151031150637, PMID: 26553010

[ref33] Korge P. Calmettes G. Weiss J. N. (2015). Increased reactive oxygen species production during reductive stress: The roles of mitochondrial glutathione and thioredoxin reductases. Biochim. Biophys. Acta 1847, 514–525. doi: 10.1016/j.bbabio.2015.02.012, PMID: 25701705 PMC4426053

[ref34] Lane N. (2009). Biodiversity: On the origin of bar codes. Nature 462, 272–274. doi: 10.1038/462272a, PMID: 19924185

[ref35] Lane N. (2011). Mitonuclear match: optimizing fitness and fertility over generations drives ageing within generations. BioEssays 33, 860–869. doi: 10.1002/bies.201100051, PMID: 21922504

[ref36] Lenth R. Singmann H. Love J. Buerkner P. Herve M. (2018). Emmeans: estimated marginal means, aka least-squares means. R Package version 1:3. doi: 10.1080/00031305.1980.10483031

[ref37] Martínez-Reyes I. Chandel N. S. (2020). Mitochondrial TCA cycle metabolites control physiology and disease. Nat. Commun. 11:102. doi: 10.1038/s41467-019-13668-3, PMID: 31900386 PMC6941980

[ref38] McDonald A. E. Pichaud N. Darveau C. A. (2018). "alternative" fuels contributing to mitochondrial electron transport: importance of non-classical pathways in the diversity of animal metabolism. Comp. Biochem. Physiol. B Biochem. Mol. Biol. 224, 185–194. doi: 10.1016/j.cbpb.2017.11.006, PMID: 29155008

[ref39] Meiklejohn C. D. Holmbeck M. A. Siddiq M. A. Abt D. N. Rand D. M. Montooth K. L. (2013). An incompatibility between a mitochondrial tRNA and its nuclear-encoded tRNA Synthetase compromises development and fitness in drosophila. PLoS Genet. 9:e1003238. doi: 10.1371/journal.pgen.1003238, PMID: 23382693 PMC3561102

[ref40] Montooth K. L. Dhawanjewar A. S. Meiklejohn C. D. (2019). Temperature-sensitive reproduction and the physiological and evolutionary potential for Mother's curse. Integr. Comp. Biol. 59, 890–899. doi: 10.1093/icb/icz091, PMID: 31173136 PMC6797906

[ref41] Mota-Martorell N. Jove M. Pradas I. Sanchez I. Gómez J. Naudi A.. (2020). Low abundance of NDUFV2 and NDUFS4 subunits of the hydrophilic complex I domain and VDAC1 predicts mammalian longevity. Redox Biol. 34:101539. doi: 10.1016/j.redox.2020.101539, PMID: 32353747 PMC7191849

[ref42] Mullen A. R. Hu Z. Shi X. Jiang L. Lindsey K. Boriack R.. (2014). Oxidation of alpha-Ketoglutarate is required for reductive carboxylation in cancer cells with mitochondrial defects. Cell Rep. 7, 1679–1690. doi: 10.1016/j.celrep.2014.04.037, PMID: 24857658 PMC4057960

[ref43] Neiman M. Taylor D. R. (2009). The causes of mutation accumulation in mitochondrial genomes. Proc. Biol. Sci. 276, 1201–1209. doi: 10.1098/rspb.2008.1758, PMID: 19203921 PMC2660971

[ref44] O'Neill S. L. Giordano R. Colbert A. M. Karr T. L. Robertson H. M. (1992). 16S rRNA phylogenetic analysis of the bacterial endosymbionts associated with cytoplasmic incompatibility in insects. Proc. Natl. Acad. Sci. U. S. A. 89, 2699–2702. 1557375 10.1073/pnas.89.7.2699 PMC48729

[ref45] Pamplona R. Jové M. Mota-Martorell N. Barja G. (2021). Is the NDUFV2 subunit of the hydrophilic complex I domain a key determinant of animal longevity? FEBS J. doi: 10.1111/febs.15714 [Epub ahead of print], PMID: 33455045

[ref46] Patel M. R. Miriyala G. K. Littleton A. J. Yang H. Trinh K. Young J. M.. (2016). A mitochondrial DNA hypomorph of cytochrome oxidase specifically impairs male fertility in Drosophila melanogaster. Elife 5:e16923. doi: 10.7554/eLife.16923, PMID: 27481326 PMC4970871

[ref47] Pichaud N. Berube R. Cote G. Belzile C. Dufresne F. Morrow G.. (2019). Age dependent dysfunction of mitochondrial and ROS metabolism induced by Mitonuclear mismatch. Front. Genet. 10:130. doi: 10.3389/fgene.2019.00130 30842791 PMC6391849

[ref48] Quinlan C. L. Perevoshchikova I. V. Hey-Mogensen M. Orr A. L. Brand M. D. (2013). Sites of reactive oxygen species generation by mitochondria oxidizing different substrates. Redox Biol. 1, 304–312. doi: 10.1016/j.redox.2013.04.005, PMID: 24024165 PMC3757699

[ref49] Rand D. M. Haney R. A. Fry A. J. (2004). Cytonuclear coevolution: the genomics of cooperation. Trends Ecol. Evol. 19, 645–653. doi: 10.1016/j.tree.2004.10.003, PMID: 16701327

[ref50] Rodrigues M. A. Martins N. E. Balancé L. F. Broom L. N. Dias A. J. S. Fernandes A. S. D.. (2015). Drosophila melanogaster larvae make nutritional choices that minimize developmental time. J. Insect Physiol. 81, 69–80. doi: 10.1016/j.jinsphys.2015.07.002, PMID: 26149766

[ref51] Rodriguez E. Hakkou M. Hagen T. M. Lemieux H. Blier P. U. (2021). Divergences in the control of mitochondrial respiration are associated With life-span variation in marine bivalves. J. Gerontol. A Biol. Sci. Med. Sci. 76, 796–804. doi: 10.1093/gerona/glaa301, PMID: 33257932

[ref52] Salminen T. S. Oliveira M. T. Cannino G. Lillsunde P. Jacobs H. T. Kaguni L. S. (2017). Mitochondrial genotype modulates mtDNA copy number and organismal phenotype in drosophila. Mitochondrion 34, 75–83. doi: 10.1016/j.mito.2017.02.001, PMID: 28214560

[ref53] Samuni Y. Goldstein S. Dean O. M. Berk M. (2013). The chemistry and biological activities of N-acetylcysteine. Biochim. Biophys. Acta 1830, 4117–4129. doi: 10.1016/j.bbagen.2013.04.016, PMID: 23618697

[ref54] Santiago J. C. Boylan J. M. Lemieux F. A. Gruppuso P. A. Sanders J. A. Rand D. M. (2021). Mitochondrial genotype alters the impact of rapamycin on the transcriptional response to nutrients in drosophila. BMC Genomics 22:213. doi: 10.1186/s12864-021-07516-2, PMID: 33761878 PMC7992956

[ref001] Simard C. J. Pelletier G. Boudreau L. H. Hebert-Chatelain E. Pichaud N. (2018). Measurement of Mitochondrial Oxygen Consumption in Permeabilized Fibers of Drosophila Using Minimal Amounts of Tissue. JoVE. 134:e57376. doi: 10.3791/57376, PMID: 29683457 PMC5933415

[ref55] Soares J. B. R. C. Gaviraghi A. Oliveira M. F. (2015). Mitochondrial physiology in the major arbovirus vector Aedes aegypti: substrate preferences and sexual differences define respiratory capacity and superoxide production. PLoS One 10:e0120600. doi: 10.1371/journal.pone.0120600, PMID: 25803027 PMC4372595

[ref56] Sweetlove L. J. Beard K. F. M. Nunes-Nesi A. Fernie A. R. Ratcliffe R. G. (2010). Not just a circle: flux modes in the plant TCA cycle. Trends Plant Sci. 15, 462–470. doi: 10.1016/j.tplants.2010.05.006, PMID: 20554469

[ref57] Taylor R. W. Turnbull D. M. (2005). Mitochondrial DNA mutations in human disease. Nat. Rev. Genet. 6, 389–402. doi: 10.1038/nrg1606, PMID: 15861210 PMC1762815

[ref58] Towarnicki S. G. Ballard J. W. O. (2017). Drosophila mitotypes determine developmental time in a diet and temperature dependent manner. J. Insect Physiol. 100, 133–139. doi: 10.1016/j.jinsphys.2017.06.002, PMID: 28619466

[ref59] Van Der Heijden E. Mcfarlane S. E. Van Der Valk T. Qvarnström A. (2019). Divergent mitochondrial and nuclear OXPHOS genes are candidates for genetic incompatibilities in Ficedula flycatchers. bioRxiv:588756. doi: 10.1101/588756

[ref60] Vander Heiden M. G. Cantley L. C. Thompson C. B. (2009). Understanding the Warburg effect: The metabolic requirements of cell proliferation. Science 324, 1029–1033. doi: 10.1126/science.1160809, PMID: 19460998 PMC2849637

[ref61] Villa-Cuesta E. Holmbeck M. A. Rand D. M. (2014). Rapamycin increases mitochondrial efficiency by mtDNA-dependent reprogramming of mitochondrial metabolism in drosophila. J. Cell Sci. 127, 2282–2290. doi: 10.1242/jcs.142026, PMID: 24610944 PMC4021473

[ref62] Vyas S. Zaganjor E. Haigis M. C. (2016). Mitochondria and cancer. Cell 166, 555–566. doi: 10.1016/j.cell.2016.07.002, PMID: 27471965 PMC5036969

[ref63] Wallace D. C. Fan W. (2010). Energetics, epigenetics, mitochondrial genetics. Mitochondrion 10, 12–31. doi: 10.1016/j.mito.2009.09.006, PMID: 19796712 PMC3245717

[ref64] Wang H. Zhao Y. Ezcurra M. Benedetto A. Gilliat A. F. Hellberg J.. (2018). A parthenogenetic quasi-program causes teratoma-like tumors during aging in wild-type *C. elegans*. NPJ Aging Mech. Dis. 4, 1–2. doi: 10.1038/s41514-018-0025-3, PMID: 29928508 PMC5998035

[ref65] Wolff J. N. Camus M. F. Clancy D. J. Dowling D. K. (2016). Complete mitochondrial genome sequences of thirteen globally sourced strains of fruit fly (Drosophila melanogaster) form a powerful model for mitochondrial research. Mitochondrial DNA A DNA Mapp. Seq. Anal. 27, 4672–4674. doi: 10.3109/19401736.2015.1106496, PMID: 26709744

[ref66] Wolff J. N. Ladoukakis E. D. Enriquez J. A. Dowling D. K. (2014). Mitonuclear interactions: evolutionary consequences over multiple biological scales. Philos. Trans. R. Soc. Lond. Ser. B Biol. Sci. 369:20130443. doi: 10.1098/rstb.2013.0443, PMID: 24864313 PMC4032519

