# Supplementary material for: Mitonuclear Interactions Produce Diverging Responses to Mild Stress in Drosophila Larvae
Source: Front Genet. 2021 Sep 16;12:734255. doi: 10.3389/fgene.2021.734255 (PMC8482813; doi:10.3389/fgene.2021.734255)
Supplement: Supplementary file 1 [file Table_1.DOCX]

| **Table S1:** Composition of the rearing and treatment media; approximate composition derived from the formulas provided by Clarke (2003), Pacheco et al. (1997), KW Alternative Feeds, and NutritionValue.Org | | | |
| --- | --- | --- | --- |
|  | **Grape juice (150 ml)** | **Control (3L)** | **High Protein (3L)** |
| **Molasses** | - | 200ml | 80ml |
| **Agar** | 15g | 24g | 24g |
| **Cornmeal** | - | 200g | 80g |
| **Yeast powder** | - | 82g | 322g |
| **Grape concentrate** | 75ml | - | - |
| **Nipagin (100 mg/L)** | 6ml | 90ml | 90ml |
| **Propionic acid** | - | 9ml | 9ml |
| **Approximate composition** | | **Control (g/L)** | **High Protein (g/L)** |
| **Water** |  | **933.33** | **933.33** |
| **Molasses** | Water | 18.15 | 7.26 |
|  | Sucrose | 30.25 | 12.10 |
|  | Glucose | 5.62 | 2.25 |
|  | Fructose | 7.35 | 2.94 |
|  | *reducing substances | 15.13 | 6.05 |
|  | Ash | 9.51 | 3.80 |
|  | Crude protein | 3.03 | 1.21 |
|  | True protein | 0.86 | 0.35 |
|  | Aconitic acid (1–5%), citric, malic, oxalic, glycolic | 3.24 | 1.30 |
|  | Mesaconic, succinic, fumaric, tartaric | 0.86 | 0.35 |
|  | **Molasses total** | **94.00** | **37.60** |
| **Agar** | C6 sugar | **8.00** | **8.00** |
| **Cornmeal** | Fat | 2.40 | 0.96 |
|  | Carbohydrate | 51.33 | 20.53 |
|  | Protein | 5.40 | 2.16 |
|  | **Cornmeal total** | **66.67** | **26.67** |
| **Yeast powder** | Protein | 13.26 | 52.07 |
|  | RNA | 2.06 | 8.07 |
|  | lipid | 0.94 | 3.69 |
|  | Ash | 2.28 | 8.94 |
|  | Carbohydrate | 8.98 | 35.27 |
|  | **Yeast total** | **27.33** | **107.33** |

**References:**

Clarke, M. A. (2003). Syrups. In *Encyclopedia of Food Sciences and Nutrition (Second Edition)* (pp. 5711-5717): Academic Press.

Pacheco, M. T. B., Caballero-Córdoba, G. M., & Sgarbieri, V. C. (1997). Composition and Nutritive Value of Yeast Biomass and Yeast Protein Concentrates. *Journal of Nutritional Science and Vitaminology, 43*(6), 601-612. doi:10.3177/jnsv.43.601

<https://www.kwalternativefeeds.co.uk/uploads/images/products/Cane%20Molasses%20Feb21.pdf>

<https://www.nutritionvalue.org/Cornmeal%2C_yellow%2C_whole-grain_nutritional_value.html>
